# Questionnaire-Based Survey on Risk Factors and Prevalence of Major Vector-Borne Diseases in the Aegean Region of Türkiye

**DOI:** 10.3390/vetsci13020114

**Published:** 2026-01-24

**Authors:** Serdar Pasa, Kerem Ural, Hasan Erdogan, Songul Erdogan, Ilia Tsachev, Mehmet Gultekin, Tahir Ozalp

**Affiliations:** 1 Department of Internal Medicine, Veterinary Faculty, Aydın Adnan Menderes University, Aydın 09100, Türkiye; spasa@adu.edu.tr (S.P.); uralkerem@gmail.com (K.U.); hasan.erdogan@adu.edu.tr (H.E.); mgultekin@adu.edu.tr (M.G.); tozalp@adu.edu.tr (T.O.); 2 Department of Clinical Science, Pleven, Faculty of Veterinary Medicine, Medical University, 5800 Pleven, Bulgaria; iliya.tsachev@trakia-uni.bg; 3 Faculty of Veterinary Medicine, Trakia University, 6000 Stara Zagora, Bulgaria; 4 Bulgarian Academy of Sciences, 1000 Sofia, Bulgaria

**Keywords:** co-infection, dog, ehrlichiosis, geographical distribution, leishmaniasis

## Abstract

This study aims to investigate the prevalence and risk factors associated with canine vector-borne diseases (CVBDs) in the Aegean Region of Türkiye. Using a questionnaire-based approach, this study intends to fill the gaps in existing knowledge regarding the prevalence and determinants of these infections. A retrospective analysis of 781 dogs presented to Aydın Adnan Menderes University Small Animal Clinic from 2019 to 2024 was conducted. Among these, 205 dogs were confirmed to have at least one CVBD using rapid diagnostic tests (SNAP 4DX PLUS and SNAP Leishmania) with confirmatory methods. Data on dog demographics, lifestyle, and environmental exposure were collected using structured questionnaires. Prevalence rates were calculated based on the at-risk population, and logistic regression determined associations between risk factors and disease occurrence. Overall CVBD prevalence was 26.3%, with Ehrlichiosis (9.9%) and Leishmaniasis (7.4%) being the most common infections. Co-infections were present in 8.3% of cases. Geographical factors significantly influenced infection rates, particularly in Aydın compared to İzmir and Muğla, while demographics like age, breed size, gender, and outdoor activity had no significant impact. This highlights the necessity for region-specific control measures and the need for consistent adherence to preventive protocols to mitigate CVBD prevalence in high-risk areas.

## Simple Summary

Vector-borne diseases are a growing concern for dog health worldwide, as they can cause severe illness and sometimes death. Understanding where and why these infections occur is essential for protecting both animals and people. This study investigates how common these diseases are in dogs living in the Aegean Region of Türkiye and which factors increase their risk. Medical records from 781 dogs examined between 2019 and 2024 were analyzed, along with information about their living conditions and outdoor exposure. Approximately one in four dogs tested positive for at least one infection, and some were infected with more than one disease at the same time. The most frequent infections were Ehrlichiosis and Leishmaniasis, both affecting the blood and immune system. Interestingly, geographic location played a larger role in disease risk than the dog’s age, breed, or daily activities. These findings highlight the importance of targeted prevention programs in high-risk areas within the Aegean Region (particularly Aydın and the surrounding districts) and underline the need for regular tick control and monitoring to reduce infection rates and protect the health of dogs and their owners.

## 1. Introduction

Canine vector-borne diseases (CVBDs) can considerably affect human and canine health status due to posing zoonotic potential [1]. A wide variety of pathogens have an etiological role in CVBDs and are all transmitted by different arthropods [2]. In this respect, Leishmaniasis, Ehrlichiosis, Dirofilariosis, Anaplasmosis, and Lyme arouse great interest in the Mediterranean [3].

CVBDs have multivarious clinical manifestations that mostly depend on the pathogenicity of the agent, asymptomatic course, existence of coinfections, settlement, and localization; these pose a challenge for the diagnosis, control, and treatment of diseases for veterinarians [4]. In addition to their veterinary importance, the worldwide spread of CVBDs has been perpetually changing as a growing global threat in recent decades [5,6,7]. The global epidemiology of canine vector-borne diseases is influenced by multiple demographic, socio-economic, anthropogenic, and environmental factors. These include changes in vector populations, globalization, international trade and transportation, expanding reservoir host populations, and climate change, all of which complicate disease control and affect prevalence patterns in both animal and public health contexts [3,8,9].

Turkey Inci et al. [10] present the existence of 19 different tick-borne diseases, with some of these carrying zoonotic potentials for humans. Moreover, they emphasize forming newly strategical control programs combatting tick-borne diseases in Turkey Associate International Health Organizations based on the One Health conception [10]. Turkey has high epidemiological importance as an endemic country for most vector borne diseases, and their distribution is prevalent, especially throughout the coastline parts, due to the geographic location and the four-season climate conditions of the country [10,11]. Nevertheless, mapping CVBDs in veterinary science as an integral part of the job is still lacking in Turkey.

In addition to Türkiye’s acknowledged epidemiological significance as an endemic region for vector-borne pathogens, various studies have yielded quantitative data regarding the prevalence of canine vector-borne diseases across different areas of the country. These studies show that *Ehrlichia canis* and *Leishmania infantum* are two of the most common pathogens found in dogs. Overall CVBD prevalence estimates are usually between 20% and 35%, but this depends on the study population and how the tests were performed [10,11,12]. Similar variability has been noted in Mediterranean and other European areas, where overall CVBD positivity rates have been reported to fluctuate between approximately 10% and over 60%, with *Ehrlichia* spp. consistently recognized as a predominant agent [8,13,14,15,16,17,18,19,20].

In the last decade, a growing number of questionnaire-based surveys in the veterinary field have been investigated related to the diagnoses, treatments, and preventions that have been applicated by veterinarians in current guidelines [21,22,23]. Some questionnaires based on veterinarian and/or owner surveys regarding canine leishmaniasis, among the common encountered CVBDs, provide data for the identification of risk evaluation maps and distributions of the disease, as well as the constitution of possible control programs in endemic areas [23,24,25]. Additionally, recent veterinarian-based questionnaire studies focusing on parasitic diseases, such as tungiasis, have further demonstrated the applicability of this approach in veterinary practice [26].

Questionnaire-based surveys based on dog owners’ options and observations can be an effective assessment tool for determining regional risk factors regarding the management and prevention of diseases. Moreover, there exists no previous questionnaire-based surveillance for CVBDs in this region. So, the objectives of this study are to obtain the first data regarding the potential individual and locational risk factors in the study area and to determine the prevalence associated with common vector-borne disease, which can enable the management and prevention of endemic diseases in the Aegean region.

## 2. Materials and Methods

### 2.1. Study Area and Study Population

Turkey is in the southeastern part of Europe and surrounded by Mediterranean, Aegean, and Black Sea [27]. Aegean Region is located western part of Turkiye, and its climate is typical Mediterranean: hot and dry summers and annual mean temperatures, relative humidity, and rainfall range from the highest 84.9 °F (29.4 °C) to the lowest 57 °F (13.9 °C), 40–60%, averaging from 75% and 400 mm to 2300 mm, respectively [28].

This single-center study was conducted at the Small Animal Clinics of the Faculty of Veterinary Medicine, Aydın Adnan Menderes University. The questionnaire was completed during routine clinical visits as part of standard anamnesis; therefore, no predefined number of expected responses was applicable.

**Figure 1:**
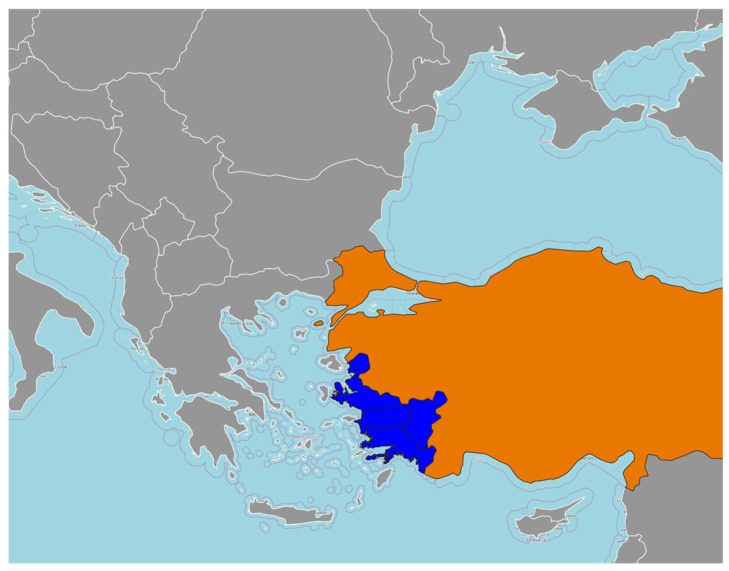
Geographical location of the study area within Europe and Türkiye, showing the Aegean Region (Aydın, Izmir, Mugla, and Denizli) where the data were collected.

In total, 781 dogs with suspected complaints were routinely submitted to the Small Animal Internal Medicine Clinics between 2019 and 2024, and of those, 205 dogs were diagnosed with CVBDs. Clinical examination of all dogs was performed, and clinical symptoms were followed with a questionnaire. This study was designed as a retrospective observational investigation based on routinely collected clinical data. No additional diagnostic or therapeutic procedures were performed for the purpose of the study. The questionnaire was administered to dog owners as part of the routine clinical intake process, and no personal identifying information was recorded. Informed consent was obtained from all owners prior to data collection. According to institutional policies, formal ethical committee approval was not required for retrospective studies based on anonymized clinical records and owner-provided information. The geographical location of the study area within Europe and Türkiye, as well as the specific region where the data were collected, is shown in Figure 1.

Dogs included in the study were presented to the Small Animal Clinics for routine clinical evaluation. The term ‘suspected complaints’ refers to clinical presentations that raised suspicion of vector-borne diseases during anamnesis and physical examination. These included, but were not limited to, lethargy, fever, weight loss, lymphadenopathy, dermatological lesions, epistaxis, anemia-related signs, thrombocytopenia-related bleeding tendencies, or unexplained chronic clinical findings.

Dogs without overt clinical signs but with epidemiological risk factors (such as history of tick exposure or residence in endemic areas) were also included when CVBD testing was deemed clinically appropriate by the attending veterinarian. Therefore, the study population represents a clinically selected group of dogs evaluated for suspected CVBDs rather than the general canine population.

### 2.2. Animal Samples Diagnosis of CVBDs

Blood samples were collected from *Vena cephalica*/*V. antebrahii* and the diagnosis of vector-borne diseases including *Diroflaria immitis*, *Anaplasma phagocytophilum*, *Anaplasma platys*, *Borrelia burgdorferi*, *Ehrlichia canis*, *Ehrlichia ewingii*, and *Leishmania infantum* were performed using the rapid-assay SNAP 4DX PLUS (IDEXX, Westbrook, ME, USA) and Snap Leishmania (IDEXX, Westbrook, ME, USA). For all dogs (n = 781) included in the study, SNAP 4Dx Plus and SNAP Leishmania tests (IDEXX, USA) were used as first-line screening tools during routine clinical evaluation, in accordance with the manufacturer’s instructions. Dogs with positive or clinically suspicious results were further evaluated using additional diagnostic methods. These confirmatory approaches included the microscopic examination of stained blood smears and/or lymph node aspirates, the indirect immunofluorescence antibody test (IFAT), and/or polymerase chain reaction (PCR), depending on the suspected pathogen and clinical presentation. Babesiosis was diagnosed based on the identification of intra-erythrocytic parasites in stained blood smears and/or PCR analysis. For the remaining investigated pathogens, confirmatory diagnostic testing was performed selectively according to the suspected agent and clinical findings. *Ehrlichia* spp. and Anaplasma *platys* infections were confirmed using the indirect immunofluorescence antibody test (IFAT) and/or PCR, whereas *Leishmania infantum* infection was confirmed using IFAT and/or PCR performed on blood or lymph node aspirates. *Borrelia burgdorferi* exposure was evaluated based on serological screening results in combination with clinical assessment. Not all dogs underwent all confirmatory diagnostic tests; additional testing was performed selectively based on initial screening results and clinical judgment. Accordingly, the diagnosis of CVBDs in this study reflects a combination of rapid screening assays supported by the pathogen-specific confirmatory methods applied in routine clinical practice.

### 2.3. The Questionnaire

A questionnaire-based survey on vector borne diseases was conducted among dog owners enrolled to Aydin Adnan Menderes University, Small Animal Clinics of Veterinary Faculty, from Aydın or the surrounding provinces. The questionnaire was administered directly by the authors during routine clinical examinations through face-to-face interviews with the dog owners. The dogs were initially evaluated based on clinical suspicion of vector-borne diseases, and diagnostic analyses were performed accordingly. For dogs with confirmed CVBD positivity, questionnaire data focusing on potential risk factors were collected during the same clinical visit. Questionnaire-based risk factor data were only collected from the owners of dogs with confirmed CVBD positivity. Accordingly, the questionnaire analyses presented in this section are based on the responses obtained from 205 dogs. The questionnaire was created in the Turkish language and was completed by the owners at the initial consultation. It consisted of 11 questions (considering the risk factors) and covered the main information related to the features of the breed and physical traits: age, gender, breed, breed type, living condition and localization, type of night shelter, habitat, observed health problems, regular health check-ups, contact with other dogs, presence/absence of tick attachment, and the management and type of the parasiticides used for prevention and control. The questionnaire was not intended as a psychometrically validated instrument; therefore, formal content validity measures such as the Content Validity Ratio (CVR) and Content Validity Index (CVI) were not applied. The questions were designed to collect descriptive clinical and epidemiological information during routine veterinary practice.

### 2.4. Prevalence

In this study, we calculated the period prevalence of CVBDs across the 2019–2024 period. The general formula for calculating period prevalence is

Prevalence (%) = [(Number of cases during the period)/(Total population at risk during the period)] × 100.

### 2.5. Statistical Analysis

Statistical analyses were conducted using IBM SPSS Statistics version 25.0 (IBM, Armonk, NY, USA). Descriptive statistics were utilized to assess the distribution of categorical variables. The normality of continuous variables was evaluated using the Shapiro–Wilk test, and non-parametric tests were applied to data that did not exhibit a normal distribution. Statistical significance was defined at *p* < 0.05.

To identify the associations between potential risk factors and infection types, stepwise forward logistic regression was utilized. Predictors demonstrating significant effects (*p* ≤ 0.05) were retained in the final models. The logistic regression models were used to estimate the odds ratios (ORs) for risk factors, including variables such as breed size, living environment, habitat, and type of parasite prevention. Wald Chi-square statistics were applied to determine the significance of each predictor. The goodness-of-fit for each model was evaluated using the Hosmer–Lemeshow test, along with the Cox and Snell R^2^ and Nagelkerke R^2^ indices.

The prevalence of vector-borne diseases was determined by calculating the proportion of positive cases among the total examined population. Confidence intervals for prevalence estimates were calculated using the Wilson score method. All statistical analyses were conducted following standard methodologies to ensure the robustness and reliability of the findings.

## 3. Results

### 3.1. Prevalence of CVBDs in the Study Population

**Figure 2:**
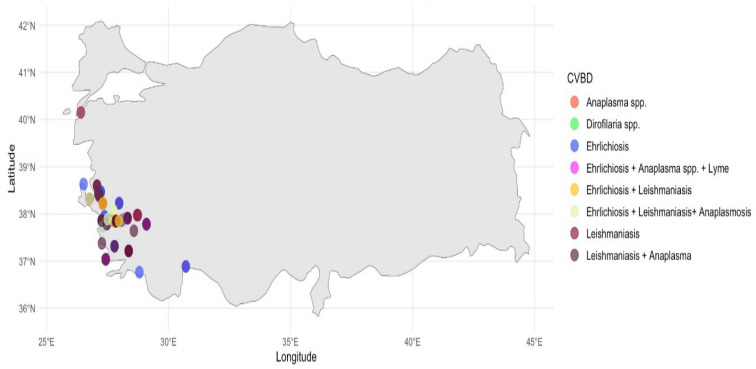
Geographical distribution map of dogs for CVBDs in Aegean Region of Türkiye, visualized Using R (the locations of the main provinces included in the analysis (Aydın, İzmir, Muğla, and Denizli) are indicated for clarity).

A total of 781 dogs were evaluated for the presence of CVBDs over a five-year period (2019–2024), with 205 dogs (26.3%) testing positive for at least one pathogen. Among the positive cases, 140 dogs were found to have mono-infection (17.9%), while 65 dogs (8.3%) presented with co-infection. The highest prevalence rates were observed for Ehrlichiosis (9.9%) and Leishmaniasis (7.4%), followed by Anaplasmosis (0.4%) and Dirofilariosis (0.3%). Furthermore, Ehrlichiosis–Anaplasmosis co-infections were notable, occurring in 3.2% of cases (Figure 2).

### 3.2. Risk Factor Assessment of CVBDs

Contrary to initial expectations, demographic factors such as age (*p* = 0.674), gender (*p* = 0.550), breed size (middle: *p* = 0.880; large: *p* = 0.746), living environment (urban vs. rural, *p* = 0.765), outdoor activity (*p* = 0.765), and coat type (*p* = 0.568) did not significantly influence the probability of co-infection. Additionally, variables such as tick exposure (*p* = 0.303) and contact with other dogs (*p* = 0.887) were found to be non-significant predictors. Purebred status was not significantly associated with the likelihood of co-infection (*p* = 0.491, OR = 0.761).

### 3.3. Geographical Variations and Risk Analysis

**Figure 3:**
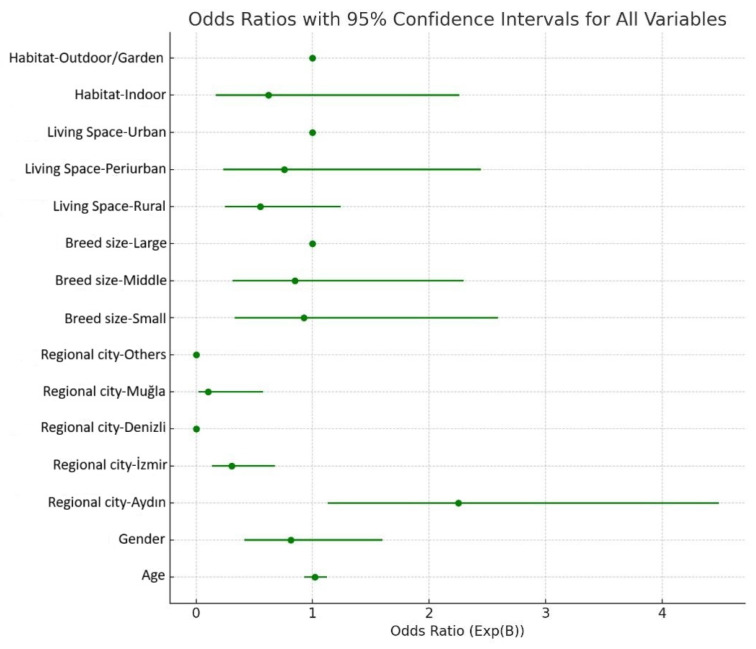
Forest plot of logistic regression analysis of some risk factors for co-infection.

In this section, geographical variation is evaluated in relation to the risk of co-infection, defined as the concurrent presence of two or more CVBDs. The geographic distribution of CVBD-positive dogs from Aydın, İzmir, Muğla, Denizli, and surrounding areas is presented in Figure 1 and Figure 2. Logistic regression analyses revealed significant regional differences in the likelihood of co-infections (Figure 3). Specifically, dogs residing in İzmir exhibited a significantly lower risk of co-infection compared to those in Aydın (OR = 0.306, *p* = 0.003), while dogs from Muğla also had a significantly reduced risk relative to the reference region included in the logistic regression model (OR = 0.104, *p* = 0.010) (Table 1).

### 3.4. Preventive Measures and Control Implications

Preventive measures against ectoparasites were widely adopted; more than half of dog owners (54.2%) reported using spot-on treatments. However, a small proportion (2.6%) reported not using any preventive agents. No statistically significant association was observed between the type of antiparasitic prevention and infection risk, including the use of combined preventive measures (*p* > 0.05).

## 4. Discussion

Recently, CVBDs are receiving growing interest worldwide due to the public health concerns for human and animals [5,6,7]. Various anthropogenic sources, including climate change, globalization, international shipping and trade, and the rapid growth of animal and reservoir host populations, have affects on worldwide distribution [3,8,9]. So, the control and monitoring of risk factors related to CVBDs is fundamental for both dog and human health [8]. The findings of the present study contribute to the understanding of CVBD epidemiology in the Aegean region by revealing distinct prevalence patterns and highlighting the relative importance of geographic location over individual dog related factors.

Questionnaire surveys in veterinary medicine enable us to assess the current state of the disease and to gather data from large geographical distribution as a feasible, cost-effective, and easy method [23,29,30]. Aydın is located at the southwestern of the Aegean Region, Turkey. The Mediterranean climatic characteristics of the Aegean region provide favorable ecological conditions for the persistence and activity of arthropod vectors, which may contribute to the sustained circulation of CVBDs in this area. In Turkey, there are a lack of investigations that point to the prevalence of vector-borne disease in dogs [10,11,12]. In a previous study conducted by some of the researchers from the team in the same region, the seroprevalence of vector-borne pathogens in dogs was assessed [12]. That study primarily focused on pathogen distribution without addressing individual and owner-related risk factors. In contrast, the present study expands upon these findings by integrating questionnaire-based data to assess potential epidemiological risk factors associated with CVBDs in the Aegean region. In another prevalence study conducted in the western part of Türkiye, the overall prevalence of CVBDs was found to be 37.1%. Among the identified pathogens, *Ehrlichia canis* exhibited the highest prevalence at 19.8%, followed by *Leishmania infantum* at 14.9%, *Anaplasma* spp. at 8.5%, and *Drofilaria immitis* at 1.2%. Additionally, the same study reported co-infection rates of 6.5% for the combinations of *Ehrlichia canis* with *Leishmania infantum* and *Ehrlichia canis* with *Anaplasma* spp. [11]. These surveys have shown similar prevalence rates for some pathogens related to CVBD in our study.

Angelou et al. [8] reported a high overall prevalence of CVBDs in dogs from Greece, with 21.8% of the sampled dogs testing seropositive for at least one pathogen. In their study, *Ehrlichia* spp. exhibited the highest seroprevalence at 12.5%, followed by *Drofilaria immitis* (9.0%) and *Anaplasma* spp. (6.2%), while *Borrelia burgdorferi* had the lowest prevalence, detected in only 0.1% of the population. These findings align with prior research conducted in various European countries, where similarly elevated prevalence rates of CVBDs were observed, despite differences in diagnostic methodologies. In regions geographically close to Türkiye, such as Bulgaria and northeastern parts of the country, studies have reported a high prevalence of CVBDs, with overall rates of 64.7% and 48.9%, respectively [13,14]. In contrast, lower prevalence rates were recorded in other parts of Europe, such as Italy and Romania, where the overall rates were 10.3% and 11.3%, respectively [15,16]. Additional studies from the Balkan Peninsula have shown CVBD prevalence ranging from 25.7% in Croatia [17] to 25.1% in Albania [18]. Furthermore, higher prevalence rates were noted in Spain (37.1%) [19] and Portugal (66%) [20]. The variations in prevalence observed in all of the studies are considered to be consistent with the levels identified in our research. Taken together, previous reports and the present findings indicate that CVBD prevalence is highly context-dependent. While similar pathogens are reported across different geographic settings, our results highlight that regional heterogeneity within the same climatic zone may substantially influence infection and co-infection dynamics, underscoring the importance of localized epidemiological assessments. These comparisons were based on geographic proximity and comparable climatic conditions, not merely on pathogen presence.

Considering the study conducted by Angelou et al. [8], the likelihood of seropositivity to *Ehrlichia* spp. was strongly linked to the lifestyle of the dogs and the antiparasitic measures taken by their owners. Their findings show that dogs living outdoors had approximately 2.3 times higher odds of being seropositive for *Ehrlichia* spp. compared to indoor dogs, likely due to their increased exposure to *Rhipicephalus sanguineus*, the main vector of this pathogen and a dominant tick species in Greece. Similarly, Selim et al. [5] found that outdoor dogs had a higher risk of being seropositive not only for *Ehrlichia* spp., but also for *Anaplasma* spp. and *Borrelia burgdorferi*, likely because of their greater exposure to various tick species, including *Rhipicephalus sanguineus*, which are competent vectors for these pathogens [31]. Furthermore, Youasa et al. [32] demonstrated that environmental factors, particularly elevation and habitat type, played a significant role in the prevalence of these infections. Their study showed that none of the dogs living at elevations above 1000 m tested positive for *Ehrlichia canis* or *Drofilaria immitis*, while seropositivity for *Ehrlichia canis* and *Anaplasma* spp. was higher in mountainous areas between 100 and 1000 m. In contrast, *Drofilaria immitis* infections were more prevalent in rural to urban environments at elevations below 100 m. Our findings align with some aspects of the previous research in terms of geographical variations influencing co-infection risk. Specifically, we observed significant regional differences in co-infection rates, with dogs from İzmir and Muğla exhibiting significantly lower odds of co-infection compared to those in Aydın relative to the reference region included in the logistic regression model (OR = 0.306, *p* = 0.003; OR = 0.104, *p* = 0.010, respectively). This suggests that, similar to the findings of Youasa et al. [32], local environmental factors such as elevation, climate, and possibly microecological conditions may play a critical role in disease transmission dynamics. Interestingly, unlike the studies by Angelou et al. [8] and Selim et al. [5], we did not observe a significant relationship between the dogs’ outdoor activity or their living environment (urban vs. rural) and co-infection risk, emphasizing the predominance of geographic factors over individual behavior or exposure patterns in our region. Given that vector-borne diseases have been transmitted through arthropods [33], someone might criticize us even if we did not discuss the proportions of diseases that we admitted in three occasionally large cities among Aegean region. Nowhere in the article did we lead readers that the same diseases in proportionally the same geographical area (Aegean region) might distinctly differ from how we observed. Given the data that vectors are ectothermic, in which bioclimatic changes (i.e., rainfall, humidity, temperature, etc.) could all influence survival growth, the special distribution of vectors, and their capacity to transmit pathogens [33], this could briefly explain the effects of climatic change (all very similar for Muğla, İzmir, Aydın, and Denizli), which did not influence our results; however, dog activity, which is the foremost culprit, could be linked to the city variations that we observed in our study. Taken together, these findings indicate that local environmental and microecological conditions may play a critical role in shaping the transmission dynamics of CVBDs, thereby contributing to the observed geographic heterogeneity within the Aegean region.

In the study by Selim et al. [5], it was observed that seropositivity to *Ehrlichia* spp. was more frequent in female dogs. However, contrasting findings have been reported in other studies, where male dogs demonstrated higher seropositivity to CVBDs, likely due to the behavioral factors that increase their exposure to vectors [34,35]. In comparison, the current study found no significant association between gender and co-infection risk (*p* = 0.550). This suggests that, at least in the regions studied, gender may not be a critical determinant of infection risk. Similarly, Selim et al. [5] noted that German Shepherds (large breed) exhibited higher seroprevalence of *Ehrlichia* spp. and *Anaplasma* spp., consistent with the known predisposition of this breed to more severe clinical manifestations of ehrlichiosis [36]. However, in the present study, breed size and specific breed characteristics did not emerge as significant factors influencing co-infection risk (*p* = 0.943). This discrepancy could reflect differences in breed distribution or environmental exposure between the study populations.

Furthermore, Hazelrig et al. [37] highlighted that older dogs had a higher risk of tick-borne pathogen infections due to longer exposure times to vectors. However, in contrast to these findings, age was not found to be a significant predictor of co-infection in the current study (*p* = 0.674). Hazelrig et al. [37] also found that dogs of larger breeds, particularly those spending more time outdoors, were at a higher risk of infection. This aligns with the general observation that larger outdoor dogs have more frequent contact with vector habitats. In contrast, our study did not find a significant relationship between outdoor activity (*p* = 0.765) or breed size (*p* = 0.943) with co-infection risk in the Aegean region.

Geographically, both studies suggest that local environmental factors can significantly influence infection dynamics [5,8]. Hazelrig et al. [37] found higher infection rates in Southern U.S. states where outdoor dogs are more prevalent, whereas the current study revealed substantial regional differences in co-infection risk within the Aegean region. Specifically, dogs residing in İzmir and Muğla had a significantly lower risk of co-infection compared to those in Aydın (OR = 0.306, *p* = 0.003; OR = 0.104, *p* = 0.010), highlighting the potential role of geographic heterogeneity in disease transmission patterns.

Both studies emphasize the importance of preventive measures. According to Hazelrig et al. [37], intact dogs were more likely to be infected with *Drofilaria immitis*, likely due to lower access to veterinary care or preventative medications [38,39]. In comparison, the current study found that the majority of dog owners adopted preventive measures (63.3% for mono-infected dogs and 79.3% for co-infected dogs). Additionally, the type of preventive measure (chemical, mechanical, or a combination) did not exhibit a notable risk for co-infection (*p* = 0.999). Atapattu et al. [40] also emphasized the need for consistent, year-round tick control in tropical regions, where the majority of owners used short-acting ectoparasiticides that required frequent administration to remain effective [41,42]. In contrast, our findings suggest that a more effective, integrated approach, combining spot-on treatments with antiparasitic collars, significantly reduced the risk of infection in high-risk areas such as Aydın. However, our study also found that not all owners followed the recommended treatment schedules, mirroring the challenges noted by Atapattu et al. [40]. This inconsistency in treatment adherence may undermine the overall effectiveness of control strategies. The efficacy of tick control products varies significantly by region. While propoxur-resistant ticks have been identified in Sri Lankan cattle [43], our study did not investigate resistance issues, but highlights the importance of continuous surveillance and adaptation of control strategies in response to local ecological conditions. Regardless of the products used, effective vector control not only requires access to reliable treatments, but also proper adherence to veterinary recommendations. This is crucial in regions like the Aegean, where local environmental factors contribute to the complexity of VBP transmission dynamics.

Although definitive diagnoses were established based on routine clinical evaluations and appropriate diagnostic testing, the use of different diagnostic modalities with varying analytical sensitivities may have introduced a degree of methodological heterogeneity. As diagnostic methods were applied according to clinical indication rather than a uniform research protocol, this aspect should be considered when interpreting the results. Therefore, prevalence estimates in this study are primarily based on serological screening results, supported by confirmatory diagnostics where clinically indicated.

## 5. Conclusions

The findings of the present study contribute to the understanding of canine vector-borne disease epidemiology in the Aegean region by demonstrating that infection and co-infection patterns are strongly influenced by geographic location rather than individual dog-related factors alone. The observed regional heterogeneity underscores the importance of localized epidemiological assessments when evaluating CVBD risk within the same climatic zone. By integrating questionnaire-based data with routine clinical diagnostics, this study highlights the value of combining owner-reported information with laboratory-confirmed outcomes to identify potential epidemiological risk factors. Such an approach may support more targeted surveillance strategies and improve preventive decision-making in endemic regions. Overall, the results emphasize the need for region-specific control and prevention programs rather than uniform strategies applied across broad geographic areas. Future studies incorporating longitudinal designs and direct environmental measurements may further clarify the mechanisms underlying geographic variation in CVBD transmission and help refine evidence-based intervention strategies. Importantly, the questionnaire-based survey approach used in this study proved to be a practical tool for capturing epidemiological information under real-world clinical conditions, complementing routine diagnostic findings and strengthening regional CVBD risk assessment.

## Figures and Tables

**Table 1:** Odds ratios and *p*-values based on likelihood ratio tests for variables associated with infection types in vector-borne diseases.

Variable		*p*-Value	Odds Ratio
Age		0.067	1.000
Gender		0.526	0.138
Breed size	Small	-	1.000
	Middle	0.880	0.924
	Large	0.746	0.849
Hair type	Short	0.346	1.155
	Long	0.421	0.982
Regional city	Aydın	0.105	1.005
	İzmir	0.003	0.306
	Denizli	0.307	1.342
	Muğla	0.010	0.104
Living space	Urban	0.000	112
	Rural	0.526	0.138
Habitat	Indoor	0.143	1.000
	Outdoor/Garden	0.236	0.875
Night shelter type	Indoor	0.218	1.432
	Outdoor	0.146	1.204
Parasite prevention	Chemical	0.236	1.112
	Mechanical	0.124	1.242
	Combination	0.067	1.522
	None	0.346	0.764

## Data Availability

The original contributions presented in this study are included in the article. Further inquiries can be directed to the corresponding author.

## Abbreviation

The following abbreviation is used in this manuscript:

CVBDs	Canine vector-borne diseases
